# Long-term effectiveness and safety of lanadelumab in Canadian patients with hereditary angioedema: a subanalysis of the EMPOWER study

**DOI:** 10.1186/s13223-025-01007-9

**Published:** 2026-01-21

**Authors:** Stephen D. Betschel, Hugo Chapdelaine, Remi Gagnon, M. Dawn Goodyear, Paul K. Keith, Ahmed El-Zoeiby, Natalie Khutoryansky, Daniel N. Castaner

**Affiliations:** 1 Department of Medicine, Division of Clinical Immunology and Allergy, University of Toronto, Toronto, ON Canada; 2 Centre Hospitalier de l’Université de Montréal, Université de Montréal, Montréal, QC Canada; 3 Montréal Clinical Research Institute, Montréal, QC Canada; 4 Clinique Spécialisée en Allergie de la Capitale, Quebec, QC Canada; 5 Section of Rheumatology, Allergy and Immunology, Department of Internal Medicine, Yale School of Medicine, New Haven, CT USA; 6 Division of Hematology and Hematologic Malignancies, Cumming School of Medicine, University of Calgary, Calgary, AB Canada; 7 Department of Medicine, Division of Clinical Immunology and Allergy, McMaster University, Hamilton, ON Canada; 8 Takeda Canada Inc., Bay Adelaide Centre, 22 Adelaide Street West, Suite 3800, Toronto, ON M5H 4E3 Canada; 9 Takeda Development Center Americas, Inc., Lexington, MA USA

**Keywords:** Effectiveness, Hereditary angioedema, Lanadelumab, Long-term prophylaxis, Real-world data, Safety, Canada

## Abstract

**Background:**

The EMPOWER Study (NCT03845400) was a phase 4, observational, non-interventional, multicenter study evaluating the real-world effectiveness and safety of lanadelumab in patients with hereditary angioedema (HAE). This subanalysis focused on lanadelumab effectiveness and safety in patients from Canada.

**Methods:**

Enrollment included patients with HAE due to C1 inhibitor deficiency. Patients were categorized as “newly treated” or “established on lanadelumab” if they had received fewer than four or at least four doses before enrollment, respectively.

**Results:**

Thirteen patients from Canada were enrolled: seven newly treated and six established on lanadelumab; these patients received lanadelumab for a mean (standard deviation [SD]) duration of 315 (224) and 536 (366) days, respectively, during the study. In newly treated patients, the mean (SD) observed HAE attack rate decreased after lanadelumab initiation from 1.5 (2.9) to 0.3 (0.6) attacks per month. In patients established on lanadelumab, the mean (SD) observed HAE attack rate was 0.1 (0.1) attacks per month throughout the study. Most HAE attacks were mild or moderate in severity (newly treated, 94%; established on lanadelumab, 100%). No study discontinuations were attributed to treatment-emergent adverse events. All treatment-emergent adverse events were non-severe, non-serious, and unrelated to lanadelumab treatment.

**Conclusions:**

Lanadelumab lowered the HAE attack rate among newly treated Canadian patients and maintained a low attack rate among those previously established on lanadelumab, demonstrating robust effectiveness across both patient groups. Effectiveness, tolerability, and safety of lanadelumab were consistent with the overall population from the EMPOWER Study, supporting the use of lanadelumab as a first-line long-term prophylactic treatment for patients with HAE in Canada.

**Trial registration:**

NCT03845400, registered February 19, 2019.

## Background

Hereditary angioedema (HAE) is a rare, and potentially life-threatening, autosomal dominant genetic disorder characterized by a deficiency in the C1 esterase inhibitor (C1INH). Mutations in the *SERPING1* gene cause decreased levels or decreased functionality of C1INH, resulting in HAE-C1INH-Type1 or HAE-C1INH-Type2 (collectively referred to as HAE-C1INH), respectively [1]. As C1INH normally functions as an important regulator of the kallikrein-kinin system to prevent excessive bradykinin production, decreased C1INH functionality leads to vasodilation and increased endothelial permeability, resulting in angioedema [1–3]. Given an estimated prevalence of 1:50,000 individuals [4] and an estimated population of 41 million people in Canada [5], about 800 Canadians are expected to be living with HAE-C1INH.

Among Canadian patients with HAE, general health has been found to be significantly reduced compared with healthy Canadians, with increased levels of bodily pain and decreased vitality [6] as well as there being increased reports of autoimmune conditions, asthma, and allergies [7]. Health-related quality of life (HRQoL) among Canadians has been negatively correlated with the number of acute HAE attacks, and as satisfaction with care and HAE control increased, so did HRQoL scores [8]. The 2019 International/Canadian HAE Guidelines, developed by the Canadian HAE Network, recommend comprehensive care for all patients to optimize treatment and outcomes including routine assessments of HRQoL [9]. This aligns with the international World Allergy Organization/European Academy of Allergy and Clinical Immunology guidelines for HAE management, which recommend that the goal of HAE treatment should be to provide total disease control to normalize patients’ lives [3]. Because HAE attacks manifest as recurrent and unpredictable edema, which can be both painful and life-threatening [2, 3], a comprehensive approach to care should provide patients with methods of disease management that reduce impacts on daily life [9].

To manage HAE, the International/Canadian HAE Guidelines recommend acute treatment to reduce attack duration and severity, short-term prophylaxis for known triggers, as well as medical and dental procedures, and long-term prophylaxis (LTP) to reduce attack frequency, severity, and duration [9]. Lanadelumab is a self-injectable, fully human monoclonal antibody that inhibits active plasma kallikrein proteolytic activity, reducing bradykinin production and providing LTP for patients with HAE [10, 11]. In 2018, lanadelumab was approved as an LTP therapy for patients with HAE in Canada and can be administered to patients 12 years of age or older [12]. Lanadelumab is also recommended by the International/Canadian Hereditary Angioedema Guidelines as a first-line LTP option in patients with HAE [9].

The EMPOWER Study (NCT03845400) was a phase 4, non-interventional, prospective, multicenter study to assess the long-term effectiveness and safety of lanadelumab among patients diagnosed with HAE in the United States and Canada in a real-world setting. Here, data from the patient cohort from Canada are reported.

## Methods

The study design for the EMPOWER Study has been described in detail previously [13]. Briefly, enrollment occurred between March 30, 2019, and October 11, 2022, for the full population, and between November 27, 2019, and December 17, 2021, for the Canadian cohort. Eligible patients had a diagnosis of HAE-C1INH, were at least 12 years old per the product labeling at study initiation, and were able to use a mobile device for data collection. Patients were termed “newly treated with lanadelumab” if they had not started lanadelumab treatment or had received fewer than four doses at enrollment. Patients “established on lanadelumab” had received at least four doses of lanadelumab before enrollment and were still receiving lanadelumab or had received their last dose within 70 days prior to enrollment. Patients were followed until death, loss to follow-up, withdrawal, or for 36 or 24 months if enrolled before or on/after September 1, 2020, respectively.

The EMPOWER Study was conducted in accordance with the International Council for Harmonization Good Clinical Practice Guidelines, ethical principles that have their origins in the Declaration of Helsinki, and other local ethical and legal requirements. Patients or their legally authorized representatives provided written informed consent prior to participation.

The primary objective, assessed in patients newly treated with lanadelumab, was to evaluate HAE attack rates before and after lanadelumab initiation in a real-world setting. Pre-enrollment HAE attack-related information was collected retrospectively by physicians using electronic case report forms (eCRFs). Throughout the study, patients self-reported HAE attack data and rescue medication in an electronic diary application for mobile phones. Physicians supplemented attack data and entered safety-related information in the eCRF at follow-up visits, which were expected to occur every 6 months.

Analyses were based on the safety population, which included all patients who received at least one dose of lanadelumab after enrollment, or the full analysis set, which included all patients in the safety population who had at least one post-baseline effectiveness outcome assessment. Lanadelumab effectiveness in patients newly treated with lanadelumab was assessed using the mean with standard deviation (SD) and median with the interquartile range (IQR) number of attacks per month for the pre-enrollment period (up to 6 months prior to enrollment) and cumulatively while receiving lanadelumab treatment. Mean reductions from the pre-lanadelumab period to the on-treatment period were calculated. Among patients established on lanadelumab, effectiveness was assessed using HAE attack rate during the overall study period.

Categorical variables are presented as proportions; continuous variables are presented using mean, SD, median, and IQR. All statistical analyses were conducted using SAS® version 9.4 (SAS Institute, Cary, NC, USA).

## Results

A total of 13 patients from five sites in Canada were enrolled in the EMPOWER Study. There were seven patients newly treated with lanadelumab and six patients established on lanadelumab (Table 1), all of whom were included in the safety set and full analysis set. Patients had a mean (SD) age of 45.6 (15.2) years: 47.9 (14.6) years in those newly treated with lanadelumab and 43.0 (16.9) years in patients established on lanadelumab. No patients were younger than 18 years of age. The majority of patients were female (9/13 [69.2%]), White (12/13 [92.3%]), and had HAE-C1INH-Type1 (12/13 [92.3%]). Most patients had a family history of HAE (9/13 [69.2%]; 1 not reported) and had not received LTP prior to lanadelumab (10/13 [76.9%]).

**Table 1:** Demographics and baseline characteristics (full analysis set)

	Patients newly treated with lanadelumab (n = 7)	Patients established on lanadelumab (n = 6)	All patients (N = 13)
Age at enrollment, years			
Mean (SD)	47.9 (14.6)	43.0 (16.9)	45.6 (15.2)
Sex, n (%)			
Female	5 (71.4)	4 (66.7)	9 (69.2)
Male	2 (28.6)	2 (33.3)	4 (30.8)
Ethnicity, n (%)			
Hispanic or Latino	0	0	0
Not Hispanic or Latino	5 (71.4)	5 (83.3)	10 (76.9)
Not reported	2 (28.6)	1 (16.7)	3 (23.1)
Race, n (%)			
White	7 (100)	5 (83.3)	12 (92.3)
Black or African American	0	1 (16.7)	1 (7.7)
Weight, kg			
n	4	2	6
Mean (SD)	85.1 (22.3)	76.1 (15.3)	82.1 (19.2)
Median (IQR)	93.6 (70.6–99.6)	76.1 (65.2–86.9)	87.8 (65.2–98.6)
BMI, kg/m^2^			
n	4	2	6
Mean (SD)	28.5 (6.2)	32.0 (10.1)	29.6 (6.8)
Median (IQR)	29.5 (23.9–33.0)	32.0 (24.8–39.1)	29.5 (24.8–34.5)
HAE-C1INH type, n (%)			
Type 1	7 (100.0)	5 (83.3)	12 (92.3)
Type 2	0	1 (16.7)	1 (7.7)
Family history of HAE,* n (%)	5 (71.4)	4 (66.7)	9 (69.2)
Prior LTP use, n (%)	3 (42.9)	0	3 (23.1)
Any medical history event,^†^ n (%)	6 (85.7)	4 (66.7)	10 (76.9)

BMI, body mass index; C1INH, C1 esterase inhibitor; HAE, hereditary angioedema; LTP, long-term prophylaxis; SD, standard deviation

*One patient did not report these data

^†^Up to 6 months prior to enrollment

The mean (SD) duration of lanadelumab use was 315 (224) days in patients newly treated with lanadelumab and 536 (366) days in patients established on lanadelumab. One patient newly treated with lanadelumab had their dosing interval extended from every 2 weeks (Q2W) to every 4 weeks (Q4W) during the study and three patients established on lanadelumab had their dosing interval extended: two patients went from Q2W to Q4W and one patient had an interim Q3W dosing period before moving to Q4W. Additionally, one patient who had been receiving lanadelumab for about 15 months started the study with Q4W dosing.

Among patients established on lanadelumab, the mean (SD) observed attack rate with treatment was 0.1 (0.1) attacks per month (median [IQR], 0.1 [0.0–0.1]). The six patients established on lanadelumab experienced a total of seven HAE attacks, with a mean (SD) duration of 26.4 (26.0) hours, all of which were mild or moderate in severity. Attacks in patients established on lanadelumab were most often treated with plasma-derived C1INH (5/7 [71.4%]); one attack was untreated (14.3%).

**Fig. 1:**
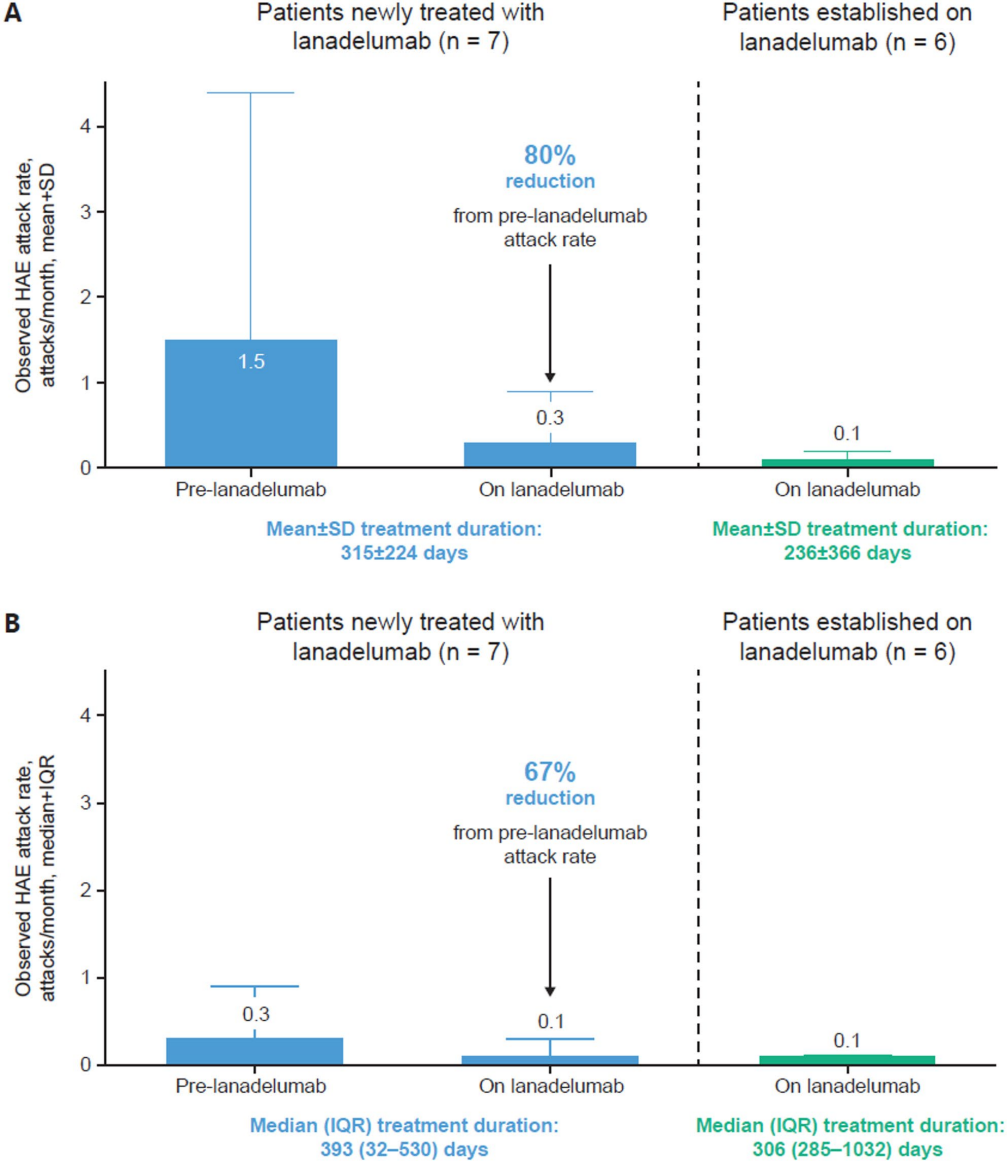
HAE attack rates among patients newly treated with and established on lanadelumab (FAS). **A** Mean observed HAE attack rates. **B** Median observed HAE attack rates. FAS, full analysis set; HAE, hereditary angioedema; IQR, interquartile range; SD, standard deviation

Among patients newly treated with lanadelumab, a low mean (SD) monthly attack rate of 0.3 (0.5) was observed within the first 69 days of lanadelumab initiation. Throughout the study, there was a mean (SD) observed attack rate of 0.3 (0.6) attacks per month, an 80% decrease from 1.5 (2.9) attacks per month prior to initiating lanadelumab (Fig. 1A). The median (IQR) observed attack rate decreased from 0.3 (0.1–0.9) to 0.1 (0.0–0.3) attacks per month (Fig. 1B). The difference between the mean and the median monthly attack rates before lanadelumab initiation in the newly treated patients was largely driven by a single patient. The seven patients newly treated with lanadelumab experienced a total of 31 HAE attacks, with a mean (SD) duration of 17.5 (15.4) hours; most of these attacks were mild or moderate (29/31 [93.5%]) (Table 2). For all attacks, patients newly treated with lanadelumab received on-demand treatment with either plasma-derived C1INH (29/31 [93.5%]) or icatibant (2/31 [6.5%]). No patients either newly treated with or established on lanadelumab required emergency room treatment for an attack during the study.

**Table 2:** Characteristics of HAE attacks on lanadelumab (full analysis set)

	Patients newly treated with lanadelumab (n = 7)	Patients established on lanadelumab (n = 6)
Number of attacks, n	31	7
Mean (SD)	4.4 (10.0)	1.2 (0.8)
Median (IQR)	1.0 (0–2)	1.0 (1–2)
Attack location, n (%)		
Peripheral	0	2 (28.6)
Abdomen	1 (3.2)	1 (14.3)
Other*	27 (87.1)	3 (42.9)
Missing	3 (9.7)	1 (14.3)
Identified or suspected trigger,^†^ n (%)		
Estrogen (including drugs)^‡^	13 (41.9)	0
Infection (including COVID-19)^§^	7 (22.6)	0
Stress^‖^	5 (16.1)	1 (14.3)
Cold^¶^	3 (9.7)	0
Other**	10 (32.3)	3 (42.9)
Missing	1 (3.2)	4 (57.1)
Healthcare encounter for the attack, n (%)		
Yes	0 (0.0)	0 (0.0)
Treatment received for the attack, n (%)		
Any treatment	31 (100.0)	6 (85.7)
Plasma-derived C1INH	29 (93.5)	5 (71.4)
Icatibant	2 (6.5)	0 (0.0)
Other	0 (0.0)	1 (14.3)^††^
HAE attack severity, n (%)		
Mild	3 (9.7)	4 (57.1)
Moderate	26 (83.9)	3 (42.9)
Severe	2 (6.5)	0 (0.0)

C1INH, C1 esterase inhibitor; HAE, hereditary angioedema; IQR, interquartile range; SD, standard deviation

*In patients newly treated with lanadelumab, all attacks in the “Other” category were reported by one patient and entered as “Abdomen, peripheral” in the attack diary. In patients established on lanadelumab, attacks in the “Other” category included “Abdomen, peripheral,” “Back,” and “Knee”

^†^Categories are non-exclusive; same attack may be listed more than once if it had at least one identified or suspected trigger

^‡^Includes triggers reported as “Cold, estrogen, food, prolonged,” “Drugs, estrogen,” “Estrogen,” “Estrogen, fighting cold,” “Estrogen, food, prolonged, stress,” “Estrogen, had COVID vaccine yesterday,” “Estrogen, infection,” “Estrogen, infection, workouts,” “Estrogen, prolonged,” and “Estrogen, stress”

^§^Includes triggers reported as “COVID, infection,” “COVID-19,” “Estrogen, fighting cold,” “Estrogen, infection,” “Estrogen, infection, workouts,” “Infection,” and “Infection, stress”

^‖^Includes triggers reported as “Estrogen, food, prolonged, stress,” “Estrogen, stress,” “Infection, stress,” “Overall stress and exhaustion,” “Stress, COVID vaccine” and “Physical, stress”

^¶^Includes triggers reported as “Cold” and “Cold, estrogen, food, prolonged”

**Includes attacks with one or more triggers other than estrogen, drugs, infection, COVID-19, stress, and/or cold

^††^The patient reported receiving lanadelumab, as it was time for their every 2 weeks treatment

Among all patients from Canada, there were no lanadelumab-related injection-site reactions reported as no patients reported treatment-emergent adverse events (TEAEs) that were related to lanadelumab LTP. No patients discontinued the study due to TEAEs and no TEAEs were severe or serious. TEAEs were reported in 3 patients, including one patient newly treated with lanadelumab who experienced a single TEAE of urticaria, classified as moderate in severity, and two patients established on lanadelumab who experienced 10 TEAEs (back pain, chest pain, COVID-19, dry eye, hyperlipidemia, nausea, procedural pain, pyrexia, thyroid mass, and osteoarthritis). Each TEAE was reported once. All TEAEs experienced by patients established on lanadelumab were mild (3/10 [30%]) or moderate (7/10 [70%]) in severity.

## Discussion

Lanadelumab has been approved for use as an LTP for patients with HAE in Canada since 2018 [12]; however, partly due to the rarity of the condition in the country, little information is available on outcomes from its use in real-world clinical practice in Canadian patients. The EMPOWER Study was a phase 4, non-interventional, self-controlled, prospective, multicenter cohort study of lanadelumab use among patients diagnosed with HAE-C1INH. In the subset of patients from Canada, including both patients newly treated with lanadelumab and established on lanadelumab, lanadelumab achieved the goals of the International/Canadian HAE Guidelines to minimize the number of HAE attacks and attack severity [9]. A low monthly HAE attack rate was observed during lanadelumab treatment; almost all attacks were mild to moderate in severity. Patients newly treated with lanadelumab experienced an 80% reduction in the mean attack rate from 1.5 attacks per month prior to initiation to 0.3 attacks per month with treatment; 94% of attacks were mild to moderate in severity. Patients established on lanadelumab experienced a mean attack rate of 0.1 HAE attacks per month during the study; all attacks were of mild to moderate severity, none were severe. No TEAEs experienced by newly treated or established patients were related to lanadelumab treatment and none were serious or severe.

Results from this subanalysis align with previously published disease control outcome data on lanadelumab use in patients with HAE. In the full analysis from the EMPOWER Study, both patients newly treated with lanadelumab and those established on lanadelumab experienced a mean monthly attack rate of 0.20, a drop from 1.42 attacks per month in patients newly treated with lanadelumab prior to initiation [13]. Similar results were also observed in the phase 3 HELP Study and HELP Open-Label Extension (OLE) Study, in which monthly attack rates were reduced to 0.26 and 0.4, respectively, after lanadelumab initiation for patients receiving 300 mg Q2W [14, 15]. Chart reviews of real-world use also show a substantial benefit for patients who receive lanadelumab. The INTEGRATED Study, a retrospective chart review of 198 patients with HAE from Europe who received lanadelumab, indicated a 95% reduction in the mean monthly attack rate from 2.98 to 0.13 [16]. Specific to Canada, a chart review among academic centers observed a 72% mean reduction in attack rates among 12 patients with HAE who received lanadelumab [17]. Additionally, although HRQoL assessments are not reported for patients in the EMPOWER Study, as they were only available for a small number of patients, significant and clinically relevant improvements in HRQoL scores were observed for patients who received lanadelumab versus placebo in the HELP Study [15]. More information on the effects of lanadelumab use on HRQoL will be important to understand the full impact of treatment on patients with HAE in Canada.

The safety results observed in this subanalysis are also consistent with the full EMPOWER Study dataset and other published lanadelumab studies [15, 18, 19]. Among patients in this subanalysis from Canada, there were no TEAEs related to lanadelumab treatment reported. All other TEAEs were mild or moderate, none were serious, and no patients discontinued treatment due to TEAEs. In the full EMPOWER Study analysis, six TEAEs observed in two patients were related to treatment, but no severe or serious TEAEs related to lanadelumab were observed, no injection-site reactions were reported, and no patients discontinued treatment due to TEAEs [13]. In the HELP and HELP OLE Studies, most TEAEs were also mild or moderate in severity, though injection-site reactions were the most commonly reported TEAE [14]. As observed here, lanadelumab treatment continues to provide a benefit for patients with HAE.

There are limitations present in this study, both due to the initial study design and due to the structure of this subanalysis. First, selection bias could affect results here due to the inclusion of patients who were already established on lanadelumab treatment and had good disease control with no adverse effects. Data collection via the self-reported mobile application and physician entry in the eCRF could also be limited by recall bias and lead to misclassification of data. No analyses of HRQoL were performed for the subset of patients from Canada due to limited data. Finally, as with all HAE studies, a low sample size is an inherent issue due to the rarity of HAE and thus also the low number of patients available for enrollment. A low sample size, as observed here with 13 patients from five centers, is particularly problematic when conducting subanalyses intended to assess effects in a specific population. Although results of this subanalysis may not be generalizable to the Canadian population as a whole, they do contribute important information to the growing body of literature on LTP use among patients with HAE in Canada. Expansion of the Canadian HAE Network Registry would increase available data on HAE in Canada and help improve the understanding of HAE treatments in real-world settings.

## Conclusion

In the EMPOWER Study, the subset of patients from Canada treated with lanadelumab experienced low rates of HAE attacks. Patients newly treated with lanadelumab had a reduced HAE attack rate compared with the attack rate prior to initiation, and most attacks were mild or moderate. Patients established on lanadelumab achieved sustained disease control with infrequent non-severe attacks. No new safety signals were identified. All TEAEs were non-severe, non-serious, and unrelated to lanadelumab, supporting the known safety and tolerability profile of lanadelumab. Results from the subset of Canadian patients in the EMPOWER Study support the use of lanadelumab as a first-line option for LTP in Canadian patients with HAE.

## Data Availability

The dataset, including the redacted study protocol, redacted statistical analysis plan, and individual participants’ data supporting the results reported in this article, will be made available within 3 months from initial request to researchers who provide a methodologically sound proposal. The data will be provided after its de-identification, in compliance with applicable privacy laws, data protection, and requirements for consent and anonymization.

## Abbreviations

C1INH: C1 esterase inhibitor
eCRF: Electronic case report form
HAE: Hereditary angioedema
HAE-C1INH-Type1/2: Hereditary angioedema caused by C1 inhibitor deficiency type 1 or 2
HRQoL: Health-related quality of life
IQR: Interquartile range
LTP: Long-term prophylaxis
OLE: Open-label extension
Q2W: Every 2 weeks
Q4W: Every 4 weeks
SD: Standard deviation
TEAE: Treatment-emergent adverse event
